# One country, two crises: what Covid-19 reveals about health inequalities among BAME communities in the United Kingdom and the sustainability of its health system?

**DOI:** 10.1186/s12939-020-01307-z

**Published:** 2020-10-27

**Authors:** Akaninyene Otu, Bright Opoku Ahinkorah, Edward Kwabena Ameyaw, Abdul-Aziz Seidu, Sanni Yaya

**Affiliations:** 1 Department of Infection and Travel Medicine, Leeds Teaching Hospitals NHS Trust, Leeds, UK; 2 Department of Internal Medicine, College of Medical Sciences, University of Calabar, Calabar, Cross Rivers State Nigeria; 3 School of Public Health, Faculty of Health, University of Technology Sydney, Liverpool, NSW Australia; 4 Department of Population and Health, College of Humanities and Legal Studies, University of Cape Coast, Cape Coast, Ghana; 5 College of Public Health, Medical and Veterinary Sciences, James Cook University, Townsville, Queensland Australia; 6 School of International Development and Global Studies, University of Ottawa, Ottawa, Ontario K1N 6N5 Canada; 7 The George Institute for Global Health, Imperial College London, London, UK

## Abstract

There has been mounting evidence of the disproportionate involvement of black, Asian and minority ethnic (BAME) communities by the Covid-19 pandemic. In the UK, this racial disparity was brought to the fore by the fact that the first 11 doctors to die in the UK from Covid-19 were of BAME background. The mortality rate from Covid-19 among people of black African descent in English hospitals has been shown to be 3.5 times higher when compared to rates among white British people. A Public Health England report revealed that Covid-19 was more likely to be diagnosed among black ethnic groups compared to white ethnic groups with the highest mortality occurring among BAME persons and persons living in the more deprived areas. People of BAME background account for 4.5% of the English population and make up 21% of the National Health Service (NHS) workforce. The UK poverty rate among BAME populations is twice as high as for white groups. Also, people of BAME backgrounds are more likely to be engaged in frontline roles. The disproportionate involvement of BAME communities by Covid-19 in the UK illuminates perennial inequalities within the society and reaffirms the strong association between ethnicity, race, socio-economic status and health outcomes. Potential reasons for the observed differences include the overrepresentation of BAME persons in frontline roles, unequal distribution of socio-economic resources, disproportionate risks to BAME staff within the NHS workspace and high ethnic predisposition to certain diseases which have been linked to poorer outcomes with Covid-19. The ethnoracialised differences in health outcomes from Covid-19 in the UK require urgent remedial measures. We provide intersectional approaches to tackle the complex racial disparities which though not entirely new in itself, have been often systematically ignored.

## COVID-19 in the UK: where the evidence leads

As Covid-19 continues to wreak havoc across the globe, there is mounting evidence that this disease is disproportionately impacting the black, Asian and minority ethnic (BAME) communities [1, 2]. In the UK, the 2011 census revealed that people of BAME background accounted for 4.5% of the English population with the Office of National Statistics estimating that it could have increased to 15.4% in 2016 [3]. However, an analysis by the Institute of Fiscal Studies published on May 1, 2020 revealed that the mortality rate from Covid-19 among people of black African descent in English hospitals was 3.5 times higher when compared to rates among white British people [4]. This disparity was not only limited to the black Africans as death rates among those of Pakistani and black Caribbean backgrounds were 2.7 and 1.7 higher respectively. This report is particularly striking as it effectively excluded age, gender and geography as possible explanations for these disparities.

It was very striking that the first 11 doctors to die in the UK from Covid-19 were of BAME background [5]. As of March 2019, there were over 1.2 million employees of the National Health Service (NHS) [6]. Of the NHS staff whose ethnicity was known, approximately 21% were of BAME background and this could be disaggregated into nursing and support staff (20%) and medical staff (44%) [7]. Emerging data shows that people of BAME backgrounds have accounted for 63% of all Covid-19 related deaths among NHS staff, 64% of deaths of nursing and support staff and 95% of deaths of medical staff [7]. Apart from this higher mortality rate, there is a growing concern that the impact of physical and social distancing measures; effectiveness of communication about Covid-19 and socio-economic fallouts from this pandemic are further disadvantaging the BAME communities in the UK.

These troubling statistics led Public Health England (PHE) to launch an investigation into the apparent disparities associated with Covid-19 in the UK. In a report published by PHE on June 2, 2020, Covid-19 was more likely to be diagnosed among black ethnic groups (486 diagnoses per 100,000 population among females and 649 in males) compared to white ethnic groups (220 per 100,000 in females and 224 in males) [8]. The death rates were highest among BAME persons and persons living in the more deprived areas; people of Bangladeshi ethnicity had twice the risk of dying compared to people of white British ethnicity [8]. There was a 10–50% greater risk of death from Covid-19 among people of Chinese, Indian, Pakistani, other Asian, Caribbean, and other ethnicities when compared to white British people [8]. This is the reverse of what was recorded in previous years, when the mortality rates were lower in BAME groups than White ethnic groups [8] for specific conditions such as chronic kidney disease. This report has caused a lot of consternation among medical and race equality organisations as it did not provide any recommendations to reduce the disparities identified [9].

### Ethnicity, genetics and disease: in search of a middle ground

Ethnicity has been previously described as a complex entity which results from the intersection of genetic make-up, behavioural patterns, social constructs and cultural identity [10]. Previous pandemics have mirrored differences in co-morbidities, immune profiles and risk of infection among people of different ethnicities [11]. Research conducted within the UK has revealed a higher predisposition to the development of coronary heart disease among people of South Asian background when compared to white Europeans [12]. Also, people of African or African Caribbean backgrounds are more predisposed to developing hypertension and having a stroke than the rest of the population who are white Europeans [12]. Type 2 diabetes is also more common among people of African, African-Caribbean and South Asian backgrounds [12]. Recent research has clearly shown poorer clinical outcomes among Covid19 patients with any co-morbidity and a correlation between a greater number of comorbidities and poorer clinical outcomes [13].

The immune system determines the body’s response to an invading pathogen such as a viral infection. It is still unclear if genes influence how our immune system responds to infections such as Covid-19 [14]. However, given the obvious racial disparities in the Covid-19 pandemic, there has been a considerable interest within the scientific community to explore genetic linkages between Covid-19 and people of ethnic minorities. An example of this is ongoing research into the role of vitamin D in predisposing persons to Covid-19 which is based on the fact that people with darker skin produce vitamin D more slowly from sunlight [14].

Some scientists have espoused that there is no genetic origin for complex conditions like heart disease or diabetes mellitus that have world-wide distribution [15]. This line of argument is supported by the limited understanding of the genetic susceptibility for many of these diseases. An alternative theory has been proposed by social epidemiologists who have promoted the role of environmental factors as key determinants of disease [15]. These environmental factors traditionally involve chemical, biological and physical agents that impinge on health but could be extended to involve social factors. Indeed, key environmental issues such as unsafe drinking water, poor sanitation & hygiene, and pollution are directly impacted by socio-economic factors [16]. These influences appear to be linked to large differentials in health within many populations and addressing inherent socio-economic equalities (which impact the environment) will be important for reducing the vulnerability of ethnic minorities. Conversely, the natural environment is crucial to socio-economic growth by providing the resources required to produce goods and services and absorbing waste products of industrialization.

## Covid-19 and health disparities: when social determinants of health take center stage

Social determinants of health influence how individuals are born, grow, live, work and age in specific environment [17]. The unequal distribution of resources, money and power at various levels influence these determinants and promote health inequities among various groups [18]. BAME communities are more likely to reside in UK’s larger cities such as London, Birmingham and Manchester and usually within densely populated wards, such as Newham and Moss Side – where Covid-19 rates are highest [4, 7]. Contact rates tend to increase with population density thus promoting the spread of directly-transmitted infectious diseases such as Covid-19. Many of these densely populated places tend to be deprived. The mortality rate from Covid-19 in the most deprived areas in the UK has been shown to be more than twice that of those in more affluent areas [19].

People of BAME backgrounds are more likely to be engaged in jobs such as public transport driving, cleaning, caring and Band 5 nursing and all these jobs cannot be done from home. The recent PHE report showed a higher death rate from Covid-19 among social care workers, nursing auxiliaries, taxi drivers, chauffeurs and security guards [8]. The higher death rate among these groups of workers may be linked to a higher risk of exposure to Covid-19 due to difficulty of implementing safe physical distancing measures in such roles [7]. In London, BAME people alone make up 67% of the adult social care workforce [7].

Poverty has been linked to late presentation for treatment in the current Covid-19 pandemic [20]. The UK poverty rate among BAME populations is twice as high as for white groups and this has been linked to factors such as higher unemployment, higher rates of economic inactivity, migration status, higher likelihood of lower pay, geographic location and lower educational attainment [21]. The higher poverty levels among BAME communities may be limiting their healthy choices be it lifestyle, nutrition or access to healthcare services.

Another contributing factor is that BAME communities in the UK tend to reside in overcrowded multigenerational households which is defined as having fewer bedrooms than needed to avoid undesirable sharing. Data from the 2011 census in the UK showed that Asian households made up 21.2% of households with multi-generational families and dependent children [22]. This data is likely to reflect South Asian cultures with tightly knit family systems in which up to three generations commonly reside under one roof [23]. Data collected between 2014 and 2017 reflected that up to 679,000 (3%) of the estimated 23 million households in England were overcrowded. However, minorities were disproportionately involved as 30% of Bangladeshi households, 16% of Pakistani households and 12% of black households were overcrowded as opposed to only 2% of white British households. In overcrowded settings, access to sources of water and sanitation facilities are likely to be limited thus promoting infectious disease transmission. Furthermore, Covid-19 control measures such as social distancing may not be easy to achieve in such settings.

Culture and language may be constituting barriers to the ability of BAME persons to access essential services. This premise is supported by the fact that BAME persons are more likely than white British persons to be born abroad [24]. There is evidence of inequities in access to NHS services with some ethnic minority groups within the UK experiencing poorer access to NHS services and poorer quality of services [25]. These inequities may be contributing to the disproportionate involvement of BAME persons in Covid-19 in the UK.

## Covid-19 is not the only virus within the United Kingdom health system: health workers from ethnic minority backgrounds in the spotlight

Data shows that BAME doctors occupy many staff grade and specialist roles which are patient-facing and invaluable to the smooth running of the NHS. There are indications that peculiarities of the NHS work environment may be predisposing staff of BAME background to Covid-19. The British Medical Association (BMA) has highlighted that BAME staff may be less empowered to speak up in settings where personal protective equipment (PPE) is lacking thus predisposing them to hazards such as Covid-19 [26]. The seeming reluctance on the part of these staff to speak up may be related to systemic discrimination. In a recent survey, up to 29% of BAME NHS staff reported being bullied or harassed by colleagues in the past 12 months [26]. Similar injustices have been reportedly meted out on BAME NHS staff by patients and other members of the public in the course of performing their duties. The BAME NHS staff also appear to be economically disadvantaged as they are systematically over-represented at lower pay bands and under-represented in the higher NHS grades. It is estimated that within the NHS, black doctors earn £10,000 and black nurses earn £2700 less annually than white colleagues [26]. These inequalities in payments, lower positions held, lack of promotion coupled with harassments are likely to demotivate the staff and cause high staff turnover ultimately resulting in poorer performance of BAME staff. This is likely to impact on the quality of care provided and eventually on the health system. This dampening effect on the health system is typical of a negative feedback loop that can exist in complex health systems. Such a feedback loop is likely to have far-reaching implications for these groups of staff that may extend to involve their dependants.

The reports of unequal Covid-19 burden among NHS staff has led to widespread fear and anxiety amongst BAME staff [27]. This fear and apprehension among BAME staff is likely to impact negatively on their willingness to provide service with untoward consequences for the NHS. In a recently conducted survey, more than three-quarters of BAME doctors expressed serious concerns about contracting Covid-19 in the course of their work citing poor access to PPE, insufficient training on how to fit masks and difficulty in accessing Covid-19 testing as reasons for these concerns [28]. With BAME staff constituting one-fifth of the NHS, the organisation has a duty of care towards these staff, and the actions taken to address the existing inequalities and inequities experienced by BAME will have significant implications for the sustainability of the UK health system. Recently, the NHS has taken concrete steps to mitigate the greater Covid-19 risk faced by BAME staff by directing trusts to perform risk assessments on these staff and implement necessary measures such as the delivery of targeted information, generation of shielding lists and adjustment of work plans to safeguard their health.

### Key approaches to close the gaps

Addressing the systemic inequities and inequalities which disadvantage BAME populations within the UK and make them prone to death from diseases such as Covid-19 is vital. The racism and structural discrimination which underlie these inequalities are complex issues and they affect different facets of BAME people’s lives. Tackling these issues by adopting a co-ordinated and intersectional approach is more likely to yield enduring results. Intersectionality, a term that was coined by Professor Kimberlé Williams Crenshaw in 1989, refers to the theoretical framework that describes how social and political identities intersect to give rise to discrimination and privilege. Fundamental issues such as economic empowerment of BAME communities can be approached by improving their access to higher-paid, better-quality jobs while providing training opportunities to endow them with the requisite knowledge and skills to fill such positions. Recruitment practices should be adjusted to reflect equity and improve the representation of BAME people in better paid jobs. The creation of ‘ethnic quotas’ for each job tier should be considered to address this problem. Diverse interview panels should be encouraged during recruitment processes. Employer-funded courses should be made available for BAME people to access career progression opportunities.

Companies and businesses should be encouraged to regularly publish aspirational targets and workforce data on race, pay bands, diversity and inclusion with a view to providing people of BAME background with equal job opportunities [29]. Free unconscious bias training resources which promote diversity should be designed and made available for all within the UK. Reverse mentoring should be encouraged to foster a deeper appreciation of equality and diversity within the workplace and society at large [29].

Within the NHS, strategies that promote inclusion and diversity in the workplace in line with Workforce Race Equality Standard should be proactively pursued. Discrimination on cultural, ethnic or religious grounds should be roundly discouraged at all levels in the workplace. Bullying and harassment should be systematically tackled within the NHS and disciplinary processes established. Effective reporting channels which guarantee confidentiality should be created to encourage people who feel bullied within the NHS to come forward and report it. Robust equity-sensitive information systems that capture equity markers regularly should be created and sustained. Innovative ways of providing regular training and career-progression opportunities to BAME staff within the NHS should be explored. Regular and comprehensive equality analysis should be carried out to evaluate the performance of the NHS.

BAME staff need to be better represented and included in high-level decision making within the NHS trusts. Staff from ethnic minorities should be supported and encouraged to voice their concerns when they find themselves in unsafe situations at work. The NHS trusts should be encouraged to treat BAME staff as “high risk and vulnerable” in regard to Covid-19. Conducting regular workplace assessments in the NHS for BAME staff to identify those at increased risk of Covid-19 is crucial. Such assessments should be matched by mitigation measures such as prioritization of BAME staff to receive infection control training such as fit testing of facemasks and redeployment of vulnerable staff away from patient-facing roles [30]. Resources for audio and visual consultations should be made available to facilitate remote working among vulnerable BAME NHS staff. Dedicated specialist counsellors and support workers should be provided to support BAME NHS staff who need to discuss anxiety or emotional issues related to the Covid-19 pandemic. Consideration should be given to prioritising BAME staff when arranging for staff testing for Covid-19.

Disaggregated data is invaluable in tailoring government’s response to pandemics such as Covid-19. Such data aid the identification of vulnerable populations and factors which impede or promote disease transmission [31]. Systems should be put in place to facilitate the acquisition and utilisation of disaggregated data during and beyond the Covid-19 pandemic. Emphasis should be placed on identifying trends related to ethnicity, occupation and the wider socioeconomic determinants. Careful statistical modelling to understand potential causal mechanisms is vital. Government has to be ready to effect rapid policy changes in response to the emerging data, particularly in a highly dynamic pandemic such as Covid-19. The benefits of adopting strategies to address any systemic inequalities that are identified is likely to extend beyond the current pandemic.

## Conclusion

Covid-19 has laid bare long-standing racial fault lines in the UK that disadvantage the already vulnerable BAME communities. The reticulated links between socioeconomic indices (income, social class, occupational background and educational achievement) and poorer health outcomes have been magnified by this pandemic. Although not novel, these issues have often been systematically ignored with catastrophic consequences. Concerted and sustained intersectional approaches are required to mitigate these deep-seated and complex ethnic inequalities.

## Data Availability

Not applicable.
